# Effects of Doping on Elastic Strain in Crystalline Ge-Sb-Te

**DOI:** 10.3390/ma18010132

**Published:** 2024-12-31

**Authors:** Ju-Young Cho, So-Yeon Lee

**Affiliations:** 1 Department of Materials Science and Engineering, Seoul National University, Seoul 08826, Republic of Korea; 2 School of Materials Science and Engineering, Kumoh National Institute of Technology, Gumi 39177, Republic of Korea

**Keywords:** PcRAM, Ge_2_Sb_2_Te_5_, amorphous phase, elastic strain energy, C-doping, N-doping, Al-doping

## Abstract

Phase-change random access memory (PcRAM) faces significant challenges due to the inherent instability of amorphous Ge_2_Sb_2_Te_5_ (GST). While doping has emerged as an effective method for amorphous stabilization, understanding the precise mechanisms of structural modification and their impact on material stability remains a critical challenge. This study provides a comprehensive investigation of elastic strain and stress in crystalline lattices induced by various dopants (C, N, and Al) through systematic measurements of film thickness changes during crystallization. Through detailed analysis of cross-sectional electron microscopy data and theoretical calculations, we reveal distinct behavior patterns between interstitial and substitutional dopants. Interstitial dopants (C and N) generate substantial elastic strain energy (~9 J/g) due to their smaller atomic radii (0.07–0.08 nm) and ability to occupy spaces between lattice sites. In contrast, substitutional dopants (Al) produce lower strain energy (~5 J/g) due to their similar atomic radius (0.14 nm) to host atoms. We demonstrate that N doping achieves higher elastic strain energy compared to C doping, attributed to its preferential formation of Ge-N bonds and resulting lattice distortions. The correlation between dopant properties, structural features, and induced strain energy provides quantitative insights for optimizing dopant selection. These findings establish a fundamental framework for understanding dopant-induced thermodynamic stabilization in GST materials, offering practical guidelines for enhancing the reliability and performance of next-generation PcRAM devices.

## 1. Introduction

The inherently metastable nature of amorphous phase-change materials significantly affects the long-term stability of phase-change random access memory (PcRAM) [1,2,3,4,5,6,7,8,9,10,11,12,13,14,15,16]. Consequently, numerous studies have focused on controlling the crystallization behavior and structural stability of amorphous phase-change materials, particularly Ge_2_Sb_2_Te_5_ (GST) [5,10,13,17,18,19,20,21,22,23,24,25,26,27,28,29,30,31,32,33,34]. Among the various approaches, doping with different elements has emerged as the most effective method for amorphous stabilization [6,8,9,18,35,36,37,38,39,40,41,42,43,44,45,46,47,48,49]. This technique improves the stability of amorphous phase-change materials through structural modification. The effectiveness of doping can be quantified by measuring the increase in crystallization temperature or activation energy through electrical or optical analysis.

Doping enables control over bond characteristics that influence crystallization kinetics. Various dopants can modify the bond networks in amorphous GST. Generally, several substances are utilized as dopants, including N [8,44,45], C [9,35,48,49], Bi [5,20], and other metals (Al [39,43], Ag [40,42], Cu [39], etc.), to attain high performance and high reliability of PcRAM. Each dopant has its own effect on the structural properties of GST; elements with relatively small atomic radii, such as C and N, occupy interstitial positions (interstitial dopants), while elements with relatively large atomic radii, such as Al and Bi, typically occupy substitutional positions (substitutional dopants) of Ge, Sb, or Te.

To describe the exact roles of the dopants, the quantitative parameters need to be addressed by considering both the kinetic and thermodynamic aspects of amorphous phase-change materials’ structural stability. As crystallization relies on the competition between the driving force (thermodynamic factor) and the atomic mobility (kinetic factor), both are important. While previous studies have primarily focused on the kinetic stability of amorphous phase-change materials [10,13,18], this study addresses the thermodynamic stabilization induced by dopants, which is related to the energy state of the amorphous state and the crystalline state of phase-change materials.

In this study, we study the fundamental question of how different dopants affect the thermodynamic stability of GST phase change through strain field modification. By combining experimental measurements of thickness changes with theoretical calculations of elastic strain energy, we reveal that the atomic radius and positioning of dopants (interstitial vs. substitutional) critically determine their effectiveness in stabilizing the amorphous phase. Our findings establish a quantitative framework for understanding dopant-induced thermodynamic stabilization, enabling more rational design of stable phase-change materials.

The innovation of this study lies in its novel approach to understanding the thermodynamic stabilization of amorphous GST through the modification of elastic strain in crystalline lattices induced by different dopants. By combining systematic experimental measurements of thickness changes during crystallization with theoretical calculations of elastic strain energy, we provide new insights into how dopants with different atomic radii and structural characteristics—such as C, N, and Al—affect the stability of GST. Unlike previous studies that primarily focus on the kinetics of crystallization, our work shifts the focus to the thermodynamic aspects, offering a deeper understanding of the stabilizing effects that different dopants have on the amorphous phase. This approach offers a more holistic view of dopant-induced phase-change behavior, advancing our ability to design and optimize phase-change materials for practical applications.

The implications of our findings extend beyond the specific case of GST. The framework we have developed for understanding dopant-induced elastic strain energy and its role in thermodynamic stabilization could have significant impacts on the broader field of material science. This model not only advances the design of more stable phase-change materials for memory devices but also provides a methodology for optimizing other types of functional materials that rely on phase transitions. In particular, the ability to engineer the thermodynamic stability of amorphous materials could be applied to a wide range of technologies, including optoelectronics and thermoelectrics, where the stability of amorphous or disordered phases is crucial. By enabling the design of materials with enhanced stability, this research has the potential to drive forward the development of next-generation devices and open up new avenues in material science research.

## 2. Materials and Methods

Amorphous Ge_2_Sb_2_Te_5_ (GST) thin films were deposited onto glass substrates using direct current (DC) or radio frequency (RF) magnetron sputtering using a Ge_2_Sb_2_Te_5_ target. Various dopants were inserted into the GST films during deposition. The individual deposition conditions for the GST films with the various dopants are listed in Table 1.

For N doping of GST, DC magnetron sputtering was used at room temperature, and the working pressure was 3 mTorr. The ambient conditions in the vacuum chamber were controlled by the flow rate of N_2_ gas (2 to 12 sccm) and Ar gas (fixed to 40 sccm) to control the N concentration in the GST thin film. For C doping of GST, RF magnetron sputtering was used at room temperature with a working pressure of 0.5 mTorr. Co-sputtering of the Ge_2_Sb_2_Te_5_ target and the C target was used. Several RF powers of the C target (11 to 89 W) with a fixed RF power of the Ge_2_Sb_2_Te_5_ target (30 W) were used to control the C concentration in the GST thin film. For the Al-doped GST, RF magnetron sputtering was used at room temperature with a working pressure of 0.5 mTorr. Co-sputtering of the Ge_2_Sb_2_Te_5_ target and the Al target was used. Several RF powers of the Al target (5 to 30 W) and a fixed RF power of the Ge_2_Sb_2_Te_5_ target (30 W) were used to control the Al concentration in the GST thin film. The concentration of dopants in the GST film was determined using Rutherford backscattering spectrometry (RBS) [Figure S1]. To measure the elastic strain during crystallization, each doped GST film was annealed at its respective crystallization temperature (170 °C for pure GST, 210 °C for Al-GST, 200 °C for C-GST, and 230 °C for N-GST). The thicknesses of the thin film samples were then observed using a field-emission scanning electron microscope (FESEM, Carl Zeiss SUPRA 55VP, Carl Zeiss AG, Oberkochen, Germany) in the cross-sectional direction. The phase changes of each doped GST film from amorphous to crystalline were determined using mechanical stress–temperature measurement, by measuring the abrupt changes in volume induced by crystallization [Figure S2 and Figure S3]. We also used Differential Scanning Calorimetry (DSC) to determine the crystallization temperatures for C- and N-doped GST [Figure S4]. The amorphous phase of the as-deposited GST films and crystalline phase of the annealed GST films were confirmed by XRD [Figure S5].

## 3. Results and Discussion

### 3.1. Thermodynamic and Kinetic Stabilization of Amorphous GST by Doping

**Figure 1:**
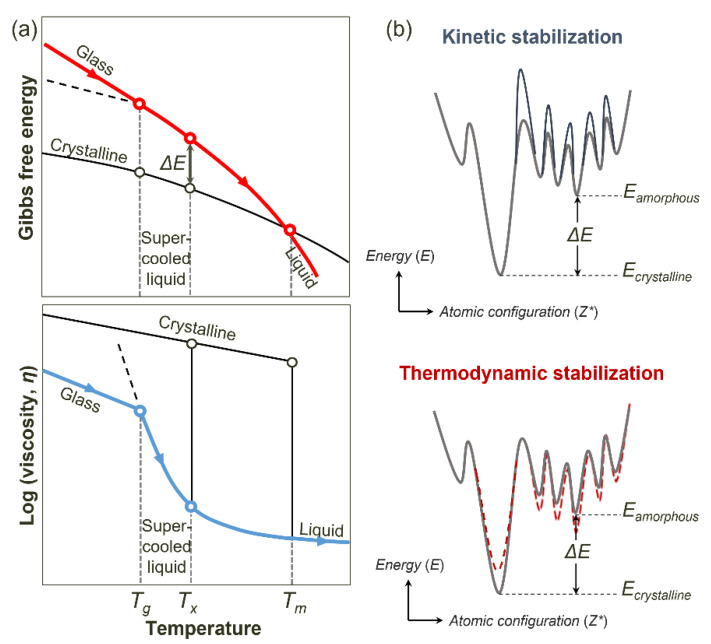
(**a**) Schematic plot showing how crystallization relies on the competition between the driving force (thermodynamic factor) and the atomic mobility (kinetic factor). (**b**) The energy landscape showing energy (*E*) as a function of the atomic configuration (Z*). *E_amorphous_* is the energy of the amorphous state, and the nearby minima are intermediate amorphous states. *E_crystalline_* is the energy of crystalline state, which has the lowest energy state. Δ*E* is the energy difference between the amorphous and crystalline states.

Figure 1a shows a schematic plot of the Gibbs free energy and the logarithm of the viscosity as a function of temperature, where *T_g_*, *T_x_*, and *T_m_* represent the glass transition temperature, the onset temperature of crystallization, and the melting temperature, respectively.

Viscosity decreases with increasing temperature and provides sufficient atomic mobility for crystallization at *T_x_*. At the same time, the difference in Gibbs free energy between the amorphous and crystalline states (Δ*E*) decreases with increasing temperature. Because Δ*E* acts as the driving force for crystallization, a lower value of Δ*E* is advantageous for stabilization of the amorphous phase. In other words, crystallization relies on the competition between the thermodynamic factor and the kinetic factor, as already mentioned.

To facilitate the discussion of the thermodynamic stabilization of amorphous structures by doping, it is useful to represent the energy of amorphous materials with different configurational features. Figure 1b illustrates two distinct stabilization mechanisms—kinetic and thermodynamic—through energy landscapes showing energy (*E*) as a function of the atomic configuration (Z*). In these landscapes, *E_amorphous_* is the energy of the amorphous state with nearby minima representing intermediate amorphous states, while *E_crystalline_* is the energy state of the crystalline state, which has the lowest energy state. Δ*E* represents the energy difference between the amorphous and crystalline states. The topology of the energy landscape is determined by the interplay between the configuration of the bond network and the energy required for atomic rearrangements, which is closely related to the fragility of the amorphous solids in the thermodynamic and kinetic factors [50]. This idea enables individual descriptions of thermodynamic and kinetic stabilization, which helps to characterize the exact role of dopants in amorphous phase-change materials.

The kinetic stabilization of amorphous materials is described by an increase in the energy barriers between intermediate minima in the energy landscape in Figure 1b. Increases in the energy barriers result in the restriction of atomic rearrangements in amorphous materials, which eventually leads to stabilization. The thermodynamic stabilization of amorphous materials is described by changes in the energy states of the amorphous and crystalline material that lead to a decrease in Δ*E*, which is related to the thermodynamic driving force for crystallization and includes (1) an increase in *E_crystalline_* and (2) a decrease in *E_amorphous_*. This case includes the stabilization of the amorphous structure (decrease in *E_amorphous_*) by decreasing *E* through the stabilization of amorphous GST by C-induced tetrahedral bonding [47,49] or an increase in the disorder in amorphous GST by Al doping [43]. However, the destabilization of the crystalline structure (increase in *E_crystalline_*) is also known to result in the relative stabilization of an amorphous structure by decreasing *E* with the N-induced strain field in the crystalline lattice of GST [37].

**Figure 2:**
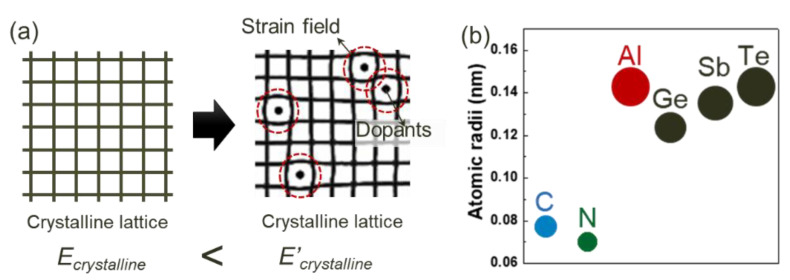
(**a**) Schematic of a distorted crystalline lattice in GST caused by a dopant-induced strain field. (**b**) Atomic radii of doping elements [35,39,43,44,45,47,48,51] compared to the those of the phase-change materials: Ge, Sb, and Te. The dots are the actual size of the atom in proportion to each other.

Figure 2a shows a schematic model of the thermodynamic destabilization of crystalline GST by a strain field in the crystalline lattice.

The left image shows an undistorted crystalline lattice with energy state *E_crystalline_*, while the right image demonstrates how dopants (marked with circles) create local distortions in the surrounding lattice (indicated by dotted regions). These dopant-induced distortions produce a compressive strain field in the crystalline lattice. This compressive strain field generates elastic strain energy in the crystalline GST films, which increases the energy state to *E’_crystalline_* (>*E_crystalline_*). As a result, the driving force for crystallization decreases (decrease in Δ*E*), leading to a relative stabilization of the amorphous state in the distorted crystalline GST.

The degree of lattice distortion caused by dopants varies depending on their atomic radii. As shown in Figure 2b, C and N have significantly smaller atomic radii (approximately 0.07–0.08 nm) compared to the constituent Ge, Sb, and Te atoms (0.12–0.15 nm), while Al has an atomic radius (approximately 0.14 nm) similar to these host atoms. Due to these size differences, C and N are categorized as interstitial dopants that occupy the spaces between lattice sites, while Al is categorized as substitutional dopant that replaces host atoms at lattice sites, as shown in Table 2.

In the following sections, the impact of doping-induced elastic strain energies in the crystalline lattices of GST films will be investigated based on the measurement of the film thickness changes associated with crystallization. The insight obtained will provide an explanation for the roles of C, N, and Al doping based on their impact on the structural modification of GST.

### 3.2. Elastic Stain and Stress Induced by Crystallization

The elastic stress Δ*σ_a–c_* induced by crystallization in a film is expressed by the strain normal to the film based on the following equation:

(1) $${\Delta \sigma }_{a-c}=-Y_{f}\frac{1-\nu }{1+\nu }\varepsilon_{zz}$$

where *Y_f_* is the biaxial modulus and is 54.9 GPa [3], and *v* is Poisson’s ratio and is 0.3399. In the calculations, the material properties described above were assumed to be the same in the pure and doped GST films [38].

The crystallization of the amorphous GST is accompanied by significant densification in the film [31]. Because the amorphous GST film is attached to the substrate, changes in the horizontal dimensions are constrained. However, the thickness is not constrained and can be regarded as a change in the elastic strain (if the plastic deformation caused by Poisson’s compression that accompanies horizontal changes is negligible). Therefore, the normal strain obtained from the thickness change is

(2) $$\varepsilon_{zz}=\frac{h-h_{0}}{h_{0}}$$

where *h* is the thickness of the film after crystallization, and *h*_0_ is the initial thickness of the film. This value can be utilized to determine the effect of doping on the elastic strain energy of amorphous GST and help verify the contribution of doping to the changes in energy states.

The decrease in *ε_zz_* with dopant concentration can be explained as follows. The strain *ε_zz_* is expected to be tensile (negative) because crystallization leads to densification and a decrease in thickness. However, when the interstitial dopants, such as C and N, distort the crystalline lattice [9,18,37,45,48,49], a compressive strain field will be produced. As a result, the tensile strain associated with crystallization will be compensated for.

**Figure 3:**
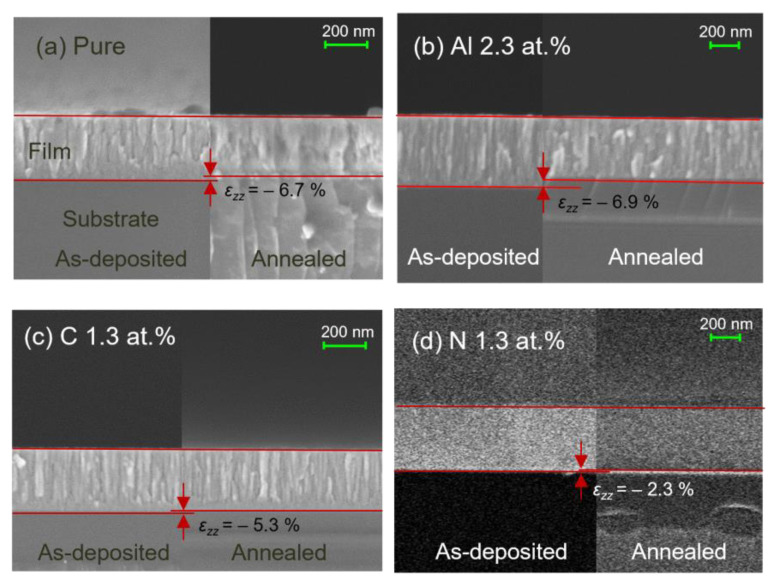
Cross-sectional FESEM images of (**a**) pure GST, (**b**) Al 2.3 at.% GST, (**c**) C 1.3 at.% GST, and (**d**) N 1.3 at.% GST before and after crystallization.

Figure 3a shows cross-sectional FESEM images of as-deposited (left) and crystallized (right) pure GST films.

**Figure 4:**
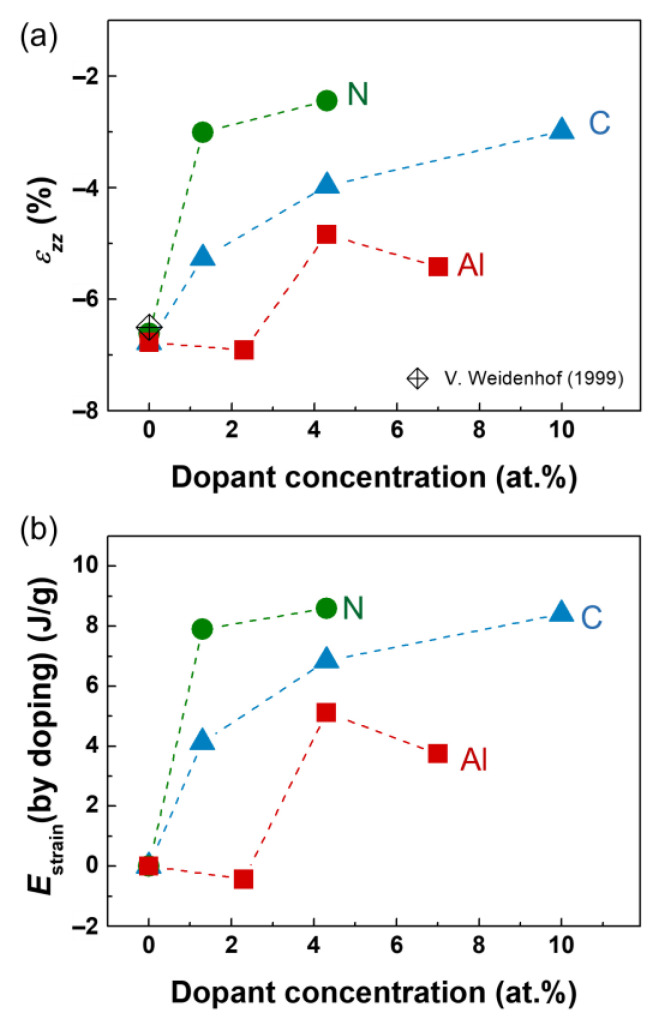
(**a**) Strain associated with changes in film thickness after crystallization for C-doped and N-doped GST [52]. (**b**) Values of Δ*E_s,doping_* for C-, N-, and Al-GST.

Figure 3b–d show images of Al 2.3 at.%, C 1.3 at.%, and N 1.3 at.% GST films, respectively. The crystallization of the samples was performed by annealing at temperatures determined for each composition as described in Materials and Methods. The thickness changes associated with crystallization were measured using the regions marked by red lines. The strains in the normal direction (*ε_zz_*) for the Al-, C-, and N-doped GST films were obtained according to Equation (2) and are shown in Figure 4.

### 3.3. Elastic Strain Energy

Figure 4a shows the strain in the normal direction induced by crystallization in C-, N-, and Al-GST. Interstitial doping (C, N) produces more compressive strain in the crystalline lattice than substitutional doping (Al). The strains associated with changes in film thickness after crystallization for C-doped and N-doped GST were utilized to calculate the dopant-induced elastic strain energy. The elastic strain energy (*E_s_*) from thickness changes in film associated with crystallization was calculated using the following equation:

*E_s_* (J/m^3^) = 0.5 *σε* (3)

By assuming elastic deformation, *ε = σ/Y* is obtained according to Hooke’s law:

*E_s_* (J/m^3^) = Δ*σ_a-c_^2^*/*Y_f_*, and *E_s_* (J/g) = Δ*σ_a-c_^2^*/*ρ*·*Y_f_*, (4)

where *ρ* is the density of the film and is 6.13 g/cm^3^ [53].

The dopant-induced elastic strain energy was determined as follows:

ΔE_s,doping_ = E_strain_(doped) − E_strain_(pure) (5)

Values of Δ*E_strain,doping_* were obtained and are shown in Figure 4b.

The values of Δ*E_strain,doping_* range from 8 to 9 J/g for N-GST, 4 to 8 J/g for C-GST, and 0 to 4 J/g for Al-GST. For similar amount of doping concentration approximately 1~2 at.%, N-GST shows the highest value of Δ*E_strain,doping_*, while Al-GST shows the lowest value.

Herein, we observed that interstitial doping (C and N) generates greater compressive strain than substitutional doping with Al. Specifically, interstitial doping produces higher elastic strain energy compared to Al-GST. This suggests that interstitial dopants induce more lattice distortion, reducing the driving force for crystallization (Δ*E*) and thereby contributing to the stabilization of the amorphous phase. This difference between interstitial and substitutional doping originates from the atomic radii of doping elements. The smaller atomic radii of C and N allow them to occupy interstitial spaces between the Ge, Sb, and Te atoms, leading to lattice distortion. In contrast, Al, with an atomic radius similar to Ge, Sb, and Te, acts as a substitutional dopant with less distortion in the crystalline lattice. These structural differences significantly influence the amount of elastic strain energy generated by doping, providing design guidance for selecting doping elements to enhance the stability of the amorphous GST.

To support the experimental data that N-GST shows higher elastic strain energy than C-GST, the difference in the bonding preferences between N and C could be considered. C has a flexible bonding nature (such as sp, sp^2^, and sp^3^ hybridizations), and it enables bonding such as Ge-C, Sb-C, and C-C [49]. This behavior may allow carbon to form bonds with Ge, Sb, and Te atoms similar to those of substitutional dopants, even though C is an interstitial dopant considering its atomic radius. In contrast, N shows a tendency to bond selectively with Ge atoms in GST. Due to the bonding preference of Ge-N in N-GST, N occupies tetrahedral interstitial site and distorts FCC unit cells, leading to the lattice parameter increases [37].

## 4. Conclusions

In this study, C-, N-, and Al-induced elastic strain and stress in crystalline lattices were investigated through measurements of the thickness changes in films associated with the crystallization process. The elastic stress induced by crystallization varies significantly with the doping elements and their concentrations; a large amount of compressive strain is produced in C- and N-doped GST, while Al doping has no significant effect on the stress change. This effect was strongly related to the structural features of the dopants. Interstitial dopants (C and N) distort the crystalline lattice and produce compressive stress and strain, while substitutional dopants (Al) have small effects on the distortion of the crystalline lattice. The quantitative relationship between dopant characteristics and elastic strain energy demonstrated in this study provides a systematic approach for evaluating dopant effectiveness in GST films. In particular, our findings that interstitial dopants generate higher elastic strain energy than substitutional dopants, and that N-doping achieves greater strain energy than C-doping due to its selective Ge-N bonding, establish clear guidelines for dopant selection. This methodology of measuring thickness changes and calculating elastic strain energy can be applied to evaluate other potential dopants and optimize GST film properties for PcRAM applications. The correlation between atomic radii, bonding preferences, and induced strain energy offers a practical framework for assessing new dopant candidates.

## Figures and Tables

**Table 1:** Sputtering conditions for the preparation of the Ge_2_Sb_2_Te_5_ films.

	Target	Power	Gas Flow
Ge_2_Sb_2_Te_5_	Ge_2_Sb_2_Te_5_	DC 80 W	Ar: 40 sccm
N doped GST	Ge_2_Sb_2_Te_5_	DC 80 W	Ar: 40 sccm N_2_: 2–12 sccm
Ge_2_Sb_2_Te_5_	Ge_2_Sb_2_Te_5_	RF 30 W	Ar: 20 sccm
C doped GST	Ge_2_Sb_2_Te_5_	RF 30 W	Ar: 20 sccm
	C	RF 11–89 W	
Al doped GST	Ge_2_Sb_2_Te_5_	RF 30 W	Ar: 20 sccm

**Table 2:** Comparison of the structural features of the dopants that are relevant to Ge_2_Sb_2_Te_5_.

	C Doping	N Doping	Al Doping
Atomic radii [nm]	0.077	0.070	0.143
Structural effect	Interstitial (Free volume occupation)		Substitutional (Atomic position)
Preferred bonds	C-Ge, C-Sb, C-C	Ge-N	Al-Sb, Al-Te

## Data Availability

The original contributions presented in the study are included in the article and Supplementary Materials; further inquiries can be directed to the corresponding authors.

## Supplementary Materials

The following supporting information can be downloaded at: https://www.mdpi.com/article/10.3390/ma18010132/s1.

## References

[1] Lencer D., Salinga M., Grabowski B., Hickel T., Neugebauer J., Wuttig M. (2008). A Map for Phase-Change Materials. Nat. Mater..

[2] Lai S. Current Status of the Phase Change Memory and Its Future. Proceedings of the IEEE International Electron Devices Meeting 2003.

[3] Chen C.-F., Schrott A., Lee M.H., Raoux S., Shih Y.H., Breitwisch M., Baumann F.H., Lai E.K., Shaw T.M., Flaitz P. Endurance Improvement of Ge2Sb2Te5-Based Phase Change Memory. Proceedings of the 2009 IEEE International Memory Workshop.

[4] Chiu Y.H., Liao Y.B., Chiang M.H., Lin C.L., Hsu W.C., Chiang P.C., Hsu Y.Y., Liu W.H., Sheu S.S., Su K.L. Impact of Resistance Drift on Multilevel PCM Design. Proceedings of the 2010 IEEE International Conference on Integrated Circuit Design and Technology, ICICDT 2010.

[5] Simandan I.-D., Sava F., Buruiana A.-T., Galca A.-C., Becherescu N., Burducea I., Mihai C., Velea A. (2021). Influence of Deposition Method on the Structural and Optical Properties of Ge2Sb2Te5. Materials.

[6] Yang T.-Y., Cho J.-Y., Park Y.-J., Joo Y.-C. (2012). Influence of Dopants on Atomic Migration and Void Formation in Molten Ge2Sb2Te5 under High-Amplitude Electrical Pulse. Acta Mater..

[7] Térébénec D., Bernier N., Castellani N., Bernard M., Jager J.-B., Tomelleri M., Paterson J., Cyrille M.-C., Tran N.-P., Giordano V.M. (2022). Innovative Nanocomposites for Low Power Phase-Change Memory: GeTe/C Multilayers. Phys. Status Solidi (RRL) Rapid Res. Lett..

[8] Park I.-M., Yang T.-Y., Jung S.W., Kim Y.K., Horii H., Joo Y.-C. (2009). Investigation of Crystallization Behaviors of Nitrogen-Doped Ge2Sb2Te5 Films by Thermomechanical Characteristics. Appl. Phys. Lett..

[9] Langhout J.D., Alverson D.N., Ginter C., Ravel B., Adams D.P., Butala M.M., Langhout J.D., Alverson D.N., Ginter C., Ravel B. (2024). Local Structure Effects of Carbon-Doping on the Phase Change Material Ge2Sb2Te5. J. Mater. Chem. C.

[10] Salinga M., Carria E., Kaldenbach A., Bornhöfft M., Benke J., Mayer J., Wuttig M. (2013). Measurement of Crystal Growth Velocity in a Melt-Quenched Phase-Change Material. Nat. Commun..

[11] Wong H.S.P., Raoux S., Kim S., Liang J., Reifenberg J.P., Rajendran B., Asheghi M., Goodson K.E. (2010). Phase Change Memory. Proc. IEEE.

[12] Khan R.S., Noor N., Jin C., Scoggin J., Woods Z., Muneer S., Ciardullo A., Ha Nguyen P., Gokirmak A., Van Dijk M. (2017). Phase Change Memory and Its Applications in Hardware Security. Security Opportunities in Nano Devices and Emerging Technologies.

[13] Wuttig M., Salinga M. (2012). Phase-Change Materials: Fast Transformers. Nat. Mater..

[14] Liu B., Wei T., Hu J., Li W., Ling Y., Liu Q., Cheng M., Song Z. (2021). Universal memory based on phase-change materials: From phase-change random access memory to optoelectronic hybrid storage. Chin. Phys. B.

[15] Raoux S. (2009). Phase Change Materials. Annu. Rev. Mater. Res..

[16] Raoux S., Xiong F., Wuttig M., Pop E. (2014). Phase Change Materials and Phase Change Memory. MRS Bull..

[17] Xiong Y., Zhang G., Tian Y., Wang J.-L., Wang Y., Zhuo Z., Zhao X. (2024). Optimization of a Ge2Sb2Te5-Based Electrically Tunable Phase-Change Thermal Emitter for Dynamic Thermal Camouflage. Materials.

[18] Cho J.-Y., Kim D., Park Y.-J., Yang T.-Y., Lee Y.-Y., Joo Y.-C. (2015). The Phase-Change Kinetics of Amorphous Ge2Sb2Te5 and Device Characteristics Investigated by Thin-Film Mechanics. Acta Mater..

[19] Wu H., Han W., Zhang X. (2022). Ultrafast Dynamics of Different Phase States Ge2Sb2Te5 Film Induced by a Femtosecond Laser Pulse Irradiation. Materials.

[20] Behrens M., Lotnyk A., Bryja H., Gerlach J.W., Rauschenbach B. (2020). Structural Transitions in Ge2Sb2Te5 Phase Change Memory Thin Films Induced by Nanosecond UV Optical Pulses. Materials.

[21] Zhu M., Cojocaru-Mirédin O., Mio A.M., Keutgen J., Küpers M., Yu Y., Cho J.-Y., Dronskowski R., Wuttig M. (2018). Unique Bond Breaking in Crystalline Phase Change Materials and the Quest for Metavalent Bonding. Adv. Mater..

[22] Kiouseloglou A., Navarro G., Sousa V., Persico A., Roule A., Cabrini A., Torelli G., Maitrejean S., Reimbold G., De Salvo B. (2014). A Novel Programming Technique to Boost Low-Resistance State Performance in Ge-Rich GST Phase Change Memory. IEEE Trans. Electron Devices.

[23] Ruitenberg G., Ruitenberg G. (2001). Crystallisation Kinetics and Optical Properties of Ge_2_Sb_2_Te_5_.

[24] Njoroge W.K., Wöltgens H.-W., Wuttig M. (2002). Density Changes upon Crystallization of Ge2Sb2.04Te4.74 Films. J. Vac. Sci. Technol. A.

[25] Lencer D., Salinga M., Wuttig M. (2011). Design Rules for Phase-Change Materials in Data Storage Applications. Adv. Mater..

[26] Zhang W., Mazzarello R., Wuttig M., Ma E. (2019). Designing Crystallization in Phase-Change Materials for Universal Memory and Neuro-Inspired Computing. Nat. Rev. Mater..

[27] Siegrist T., Jost P., Volker H., Woda M., Merkelbach P., Schlockermann C., Wuttig M. (2011). Disorder-Induced Localization in Crystalline Phase-Change Materials. Nat. Mater..

[28] Yeo E.G., Shi L.P., Zhao R., Chong T.C. (2006). Investigation on Ultra-High Density and High Speed Non-Volatile Phase Change Random Access Memory (PCRAM) by Material Engineering. MRS Online Proc. Libr..

[29] Wuttig M., Bhaskaran H., Taubner T. (2017). Phase-Change Materials for Non-Volatile Photonic Applications. Nat. Photonics.

[30] Wuttig M., Yamada N. (2007). Phase-Change Materials for Rewriteable Data Storage. Nat. Mater..

[31] Siegel J., Schropp A., Solís Céspedes J., Afonso C.N., Wuttig M. (2004). Rewritable Phase-Change Optical Recording in Ge₂Sb₂Te₅ FIlms Induced by Picosecond Laser Pulses. Appl. Phys. Lett..

[32] Friedrich I., Weidenhof V., Njoroge W., Franz P., Wuttig M. (2000). Structural Transformations of Ge2Sb2Te5 Films Studied by Electrical Resistance Measurements. J. Appl. Phys..

[33] Wuttig M., Lüsebrink D., Wamwangi D., Wełnic W., Gilleßen M., Dronskowski R. (2007). The Role of Vacancies and Local Distortions in the Design of New Phase-Change Materials. Nat. Mater..

[34] Wuttig M. (2005). Towards a Universal Memory?. Nat. Mater..

[35] Hubert Q., Jahan C., Toffoli A., Navarro G., Chandrashekar S., Noe P., Blachier D., Sousa V., Perniola L., Nodin J.-F. Lowering the Reset Current and Power Consumption of Phase-Change Memories with Carbon-Doped Ge2Sb2Te5. Proceedings of the 2012 4th IEEE International Memory Workshop.

[36] Song K.-H., Kim S.-W., Seo J.-H., Lee H.-Y. (2008). Characteristics of Amorphous Ag0.1(Ge2Sb2Te5)0.9 Thin Film and Its Ultrafast Crystallization. J. Appl. Phys..

[37] Jeong T.H., Kim M.R., Seo H., Park J.W., Yeon C. (2000). Crystal Structure and Microstructure of Nitrogen-Doped Ge2Sb2Te5 Thin Film—IOPscience. Jpn. J. Appl. Phys..

[38] Kim S.-J., Lee S.-C., Choi J.-H. (2012). Density Functional Calculations on the Mechanical Properties of Nitrogen or Oxygen Doped Crystalline Ge2Sb2Te5. J. Nanosci. Nanotechnol..

[39] Raoux S., Salinga M., Jordan-Sweet J.L., Kellock A. (2007). Effect of Al and Cu Doping on the Crystallization Properties of the Phase Change Materials SbTe and GeSb. J. Appl. Phys..

[40] Zhou J., Sun Z., Xu L., Ahuja R. (2008). Effect of Dopants on the Structure and Properties of Ge2Sb2Te5 Studied by Ab Initio Calculations. Solid State Commun..

[41] Chen S.F., Chen J.K., Chen T.P. (2008). Effects of Bi on Crystallisation in Ge–Sb–Te–Bi. Mater. Sci. Technol..

[42] Lie C.-T., Kuo P.-C., Hsu W.-C., Wu T.-H., Chen P.-W., Chen S.-C. (2003). Ge2Sb2Te5 Thin Film Doped with Silver—IOPscience. Jpn. J. Appl. Phys..

[43] Wang G., Shen X., Nie Q., Wang R., Wu L., Lv Y., Chen F., Fu J., Dai S., Li J. (2012). Improved Thermal and Electrical Properties of Al-Doped Ge2Sb2Te5 Films for Phase-Change Random Access Memory. J. Phys. D Appl. Phys..

[44] Dimitrov D., Shieh H.-P.D. (2004). The Influence of Oxygen and Nitrogen Doping on GeSbTe Phase-Change Optical Recording Media Properties. Mater. Sci. Eng. B.

[45] Park I.-M., Cho J.-Y., Yang T.-Y., Park E.S., Joo Y.-C. (2011). Thermomechanical Analysis on the Phase Stability of Nitrogen-Doped Amorphous Ge2Sb2Te5 Films—IOPscience. Jpn. J. Appl. Phys..

[46] Li C., Song H., Dai Z., Zhao Z., Liu C., Yang H., Cui C., Miao L. (2022). High Thermoelectric Performance Achieved in Sb-Doped GeTe by Manipulating Carrier Concentration and Nanoscale Twin Grains. Materials.

[47] Cho E., Han S., Kim D., Horii H., Nam H.-S. (2011). Ab Initio Study on Influence of Dopants on Crystalline and Amorphous Ge2Sb2Te5. J. Appl. Phys..

[48] Cho E., Youn Y., Han S. (2011). Enhanced Amorphous Stability of Carbon-Doped Ge2Sb2Te5: Ab Initio Investigation. Appl. Phys. Lett..

[49] Borisenko K.B., Chen Y., Cockayne D.J.H., Song S.A., Jeong H.S. (2011). Understanding Atomic Structures of Amorphous C-Doped Ge2Sb2Te5 Phase-Change Memory Materials. Acta Mater..

[50] Angell C.A. (1991). Relaxation in Liquids, Polymers and Plastic Crystals—Strong/Fragile Patterns and Problems. J. Non-Cryst. Solids.

[51] Hubert Q., Jahan C., Toffoli A., Navarro G., Chandrashekar S., Noé P., Sousa V., Perniola L., Nodin J.-F., Persico A. Carbon-Doped Ge2Sb2Te5 Phase-Change Memory Devices Featuring Reduced RESET Current and Power Consumption. Proceedings of the 2012 European Solid-State Device Research Conference (ESSDERC).

[52] Weidenhof V., Friedrich I., Ziegler S., Wuttig M. (1999). Atomic Force Microscopy Study of Laser Induced Phase Transitions in Ge2Sb2Te5. J. Appl. Phys..

[53] Nonaka T., Ohbayashi G., Toriumi Y., Mori Y., Hashimoto H. (2000). Crystal Structure of GeTe and Ge2Sb2Te5 Meta-Stable Phase. Thin Solid Film..

