# Supplementary material for: Effects of Doping on Elastic Strain in Crystalline Ge-Sb-Te
Source: Materials (Basel). 2024 Dec 31;18(1):132. doi: 10.3390/ma18010132 (PMC11722036; doi:10.3390/ma18010132)
Supplement: Supplementary file 1 [file materials-18-00132-s001.zip › materials-3308773-supplementary.pdf]

Supplementary Materials

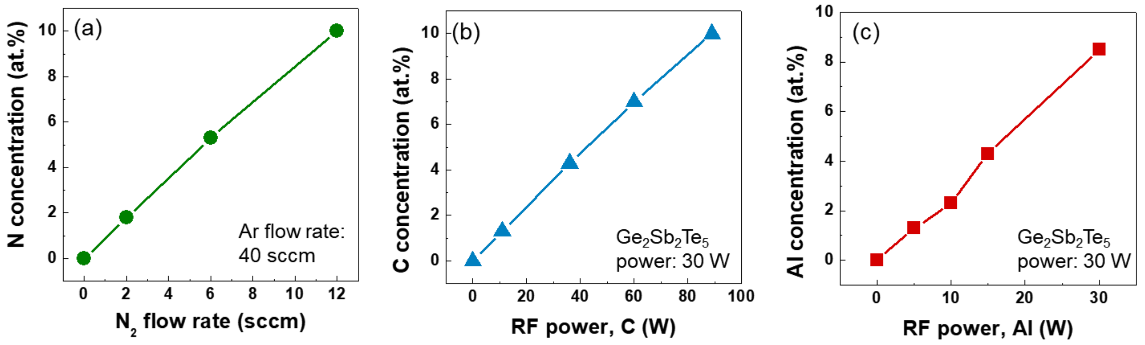

Figure S1 Dopant concentrations in the $Ge_2Sb_2Te_5$ films as a function of deposition conditions detected by Rutherford backscattering spectrometry (RBS). (a) N concentration as a function of the N_2 flow rate. (b) C concentration as a function of the RF power of the C target. (c) Al concentration as a function of the RF power of the Al target.

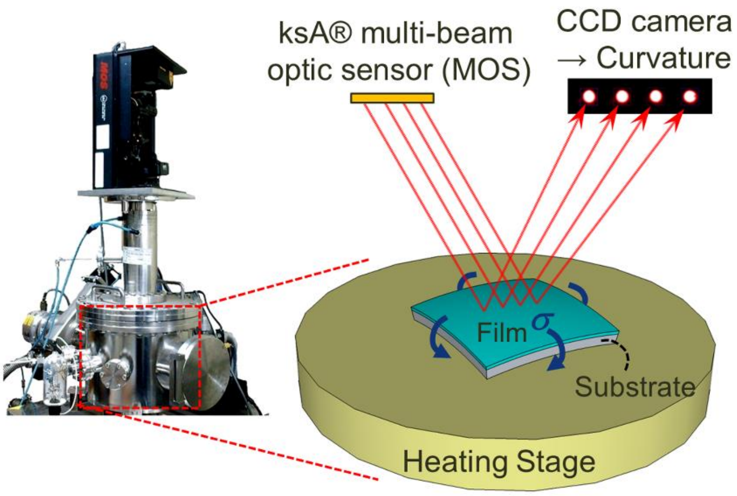

Figure S2 Real-time substrate curvature measurement using a multi-beam optical sensor (MOS) set up in the laboratory. The curvature is determined from the beam spacing, and the biaxial stress of the film was then determined from the changes in curvature according to the Stoney equation.

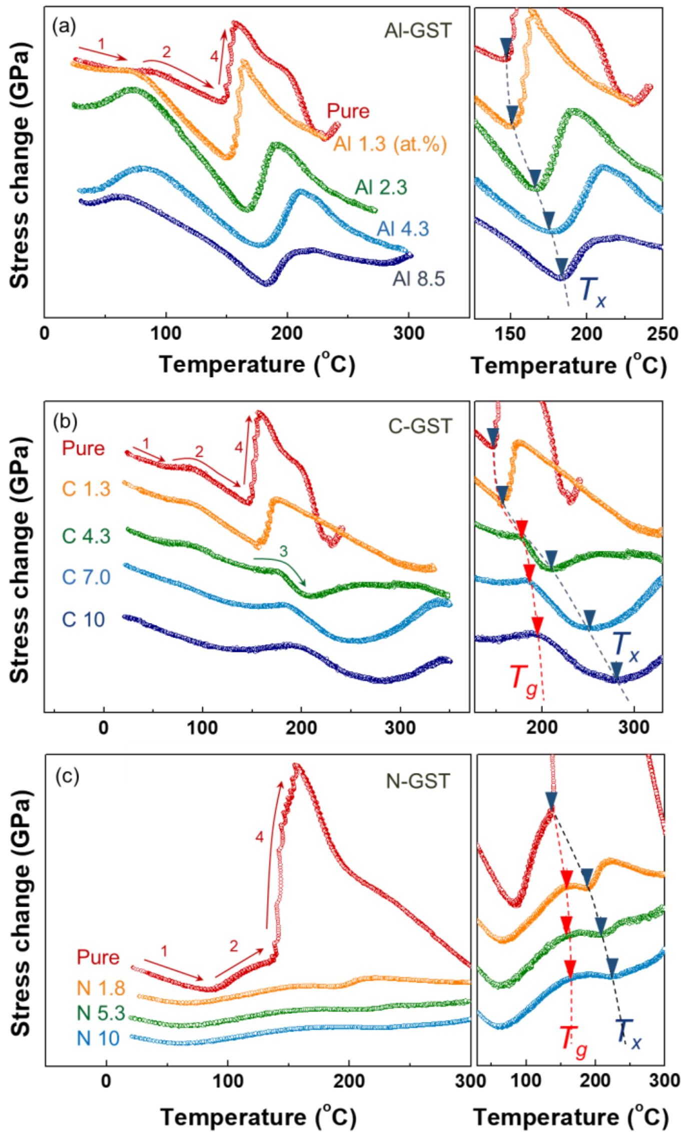

Figure S3 Stress-temperature curves of (a) Al-doped and (b) C-doped and (c) N-doped GST films during heating. Magnified views are shown on the right side of each graph (arrows indicate T_x, crystallization temperature and T_g, glass transition temperature). Stress changes associated with thermal expansion, structural relaxation and crystallization are denoted as 1, 2 and 4, respectively.

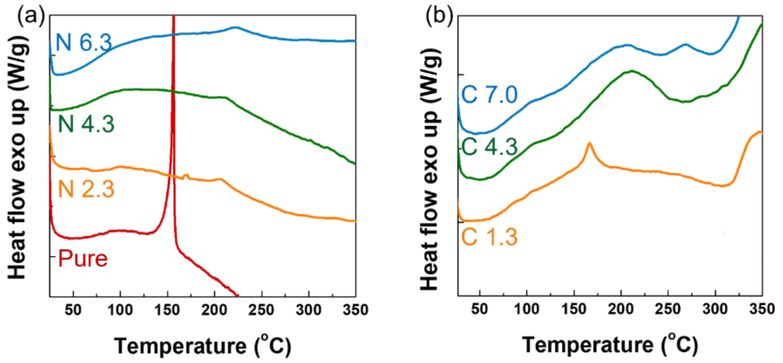

Figure S4 (a) DSC profiles of (a) N-doped GST and (b) C-doped GST, the peaks indicate the crystallization temperatures.

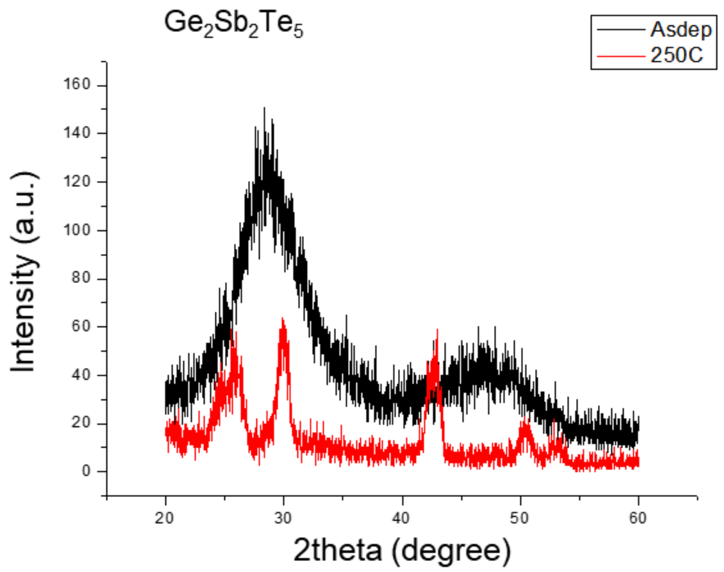

Figure S5 XRD data of GST films. The as-deposited GST film is amorphous phase.
